# Genetic Code Expansion and a Photo-Cross-Linking Reaction Facilitate Ribosome Display Selections for Identifying a Wide Range of Affinity Peptides

**DOI:** 10.3390/ijms242115661

**Published:** 2023-10-27

**Authors:** Takuto Furuhashi, Kensaku Sakamoto, Akira Wada

**Affiliations:** 1 Graduate School of Medical Life Science, Yokohama City University, 1-7-29 Suehiro-cho, Tsurumi-ku, Yokohama 230-0045, Kanagawa, Japan; 2 Laboratory for Advanced Biomolecular Engineering, RIKEN Center for Biosystems Dynamics Research, 1-7-22 Suehiro-cho, Tsurumi-ku, Yokohama 230-0045, Kanagawa, Japan; 3 Laboratory for Nonnatural Amino Acid Technology, RIKEN Center for Biosystems Dynamics Research, 1-7-22 Suehiro-cho, Tsurumi-ku, Yokohama 230-0045, Kanagawa, Japan; mnm5u.sakamoto@gmail.com; 4 Department of Drug Target Protein Research, School of Medicine, Shinshu University, 3-1-1 Asahi, Matsumoto 390-8621, Nagano, Japan

**Keywords:** in vitro translation, ribosome display, affinity peptide, genetic code expansion, photo-cross-linking reaction

## Abstract

Cell-free molecular display techniques have been utilized to select various affinity peptides from peptide libraries. However, conventional techniques have difficulties associated with the translational termination through in-frame UAG stop codons and the amplification of non-specific peptides, which hinders the desirable selection of low-affinity peptides. To overcome these problems, we established a scheme for ribosome display selection of peptide epitopes bound to monoclonal antibodies and then applied genetic code expansion with synthetic X-tRNA^UAG^ reprogramming of the UAG codons (X = Tyr, Trp, or *p*-benzoyl-l-phenylalanine (*p*Bzo-Phe)) to the scheme. Based on the assessment of the efficiency of in vitro translation with X-tRNA^UAG^, we carried out ribosome display selection with genetic code expansion using Trp-tRNA^UAG^, and we verified that affinity peptides could be identified efficiently regardless of the presence of UAG codons in the peptide coding sequences. Additionally, after evaluating the photo-cross-linking reactions of *p*Bzo-Phe-incorporated peptides, we performed ribosome display selection of low-affinity peptides in combination with genetic code expansion using *p*Bzo-Phe-tRNA^UAG^ and photo-irradiation. The results demonstrated that sub-micromolar low-affinity peptide epitopes could be identified through the formation of photo-induced covalent bonds with monoclonal antibodies. Thus, the developed ribosome display techniques could contribute to the promotion of diverse peptide-based research.

## 1. Introduction

A ribosome, bio-machinery consisting of protein and RNA molecules [1,2,3], takes up 20 types of canonical amino acid-charged tRNAs (AA-tRNAs) in response to the genetic information of mRNAs to facilitate peptide elongation reactions. Ribosome-driven translation is essential for the synthesis of a wide variety of proteins that control cellular activity in the spatiotemporal manner. Thus, understanding the relationship between the structure and function of ribosomes has been a focus of attention in the life sciences [4,5,6]. In parallel, cell-free protein synthesis systems involving ribosomes have been developed using lysates of wheat germ, rabbit reticulocytes, insects, and *Escherichia coli* [7,8,9,10,11]. Characteristically, these systems have enabled the production of not only single-type proteins but also diverse peptides in small tubes within a few hours without specialized equipment. Therefore, they have been successfully applied in the manufacturing of transmembrane proteins and therapeutic biomolecules, fabrication of protein arrays for high-throughput proteomic analysis, and engineering of metabolic pathways and genetic circuits [12,13,14].

Additionally, molecular display techniques coupled with cell-free protein synthesis systems have been used to obtain new affinity peptides that interact with target molecules in peptide libraries [15,16,17]. A vital feature of these techniques is the use of phenotype-genotype complexes that link peptides to their corresponding genes. For instance, in the ribosome display technique [18,19,20,21], a pool of peptide-ribosome-mRNA complexes is constructed by attaching a nascent peptide to its mRNA on the ribosome. In the mRNA display technique [16,22,23,24], a pool of peptide-puromycin-mRNA complexes is synthesized by linking the C-terminus of a nascent peptide in the ribosome and a puromycin modified at the 3′ terminus of an mRNA. These transient complexes are indispensable for screening peptide libraries and deciphering sequences of affinity peptides bound to target molecules. Moreover, as opposed to the phage display technique, which suffers limitations derived from the use of living *E. coli* cells [17,25,26,27], cell-free molecular display techniques allow the use of desirable peptide libraries with higher diversity. This feature is advantageous for exploring previously unknown affinity peptides. However, some negative aspects require their performances to be improved.

DNA constructs coding combinatorial peptides are transcribed into an mRNA pool in vitro and then translated into a peptide library to be screened using molecular display techniques. NNK codons (N = A, G, C, or T; K = G or T) are commonly used to constitute the combinatorial peptide-coding sequences along with minimizing the occurrence of stop codons in the coding frame. However, this method cannot avoid the appearance of in-frame TAG amber stop codons in the initial DNA sequences. Thus, translational termination by the amber stop codons causes a reduction in the diversity of peptide libraries. In the conventional selection of affinity peptides against partner molecules, another drawback is that nonspecific peptides are often amplified through multiple selection rounds due to several causes (such as insufficient washing or a small number of partner molecules), preventing the identification of desired peptides. It is also difficult to obtain low-affinity peptides from peptide libraries even though they are specific binders to partner molecules. Therefore, the development of an advanced molecular display technique is required to overcome these difficulties.

To maintain the diversity of the designed peptide libraries and identify low-affinity peptides that interact specifically with partner molecules, we aimed to incorporate the advantages of genetic code expansion and photo-cross-linking reactions into ribosome display selections. Genetic code expansion allows the translation of the UAG amber stop codon into canonical or non-canonical amino acids (designated as X) with synthetic X-charged tRNA reading the UAG codon (X-tRNA^UAG^) [28,29,30,31,32,33]. Reprogramming of the UAG codon could suppress immature translational termination at the positions of undesirable in-frame UAG codons. Moreover, using genetic code expansion with X-tRNA^UAG^, a photo-cross-linkable non-canonical amino acid could be assigned to the UAG positions in peptide coding sequences [34,35], allowing the formation of photo-induced covalent bonds between affinity peptides and their partner molecules. Thus, the incorporation of photo-cross-linkable non-canonical amino acids into a peptide library would be useful for identifying low-affinity peptides covalently bound to partner molecules through selections after photo-irradiation.

In the present study, we developed ribosome display techniques for identifying affinity peptides as peptide epitopes binding to monoclonal antibodies and verified the utility of genetic code expansion using X-tRNA^UAG^ for improving ribosome display selections. Since facile and robust techniques for revealing the sequences of peptide epitopes recognized by developed monoclonal antibodies are of bio-pharmaceutical and clinical importance [36,37,38], we originally constructed and assessed a scheme for ribosome display selections to identify various peptide epitopes that interact with monoclonal antibodies as partner molecules. Additionally, based on the assessment of the efficiency of cell-free protein expression in the presence of synthetic Tyr-tRNA^UAG^ and Trp-tRNA^UAG^, we incorporated genetic code expansion using Trp-tRNA^UAG^ into the established scheme and certified that affinity peptides could be identified through ribosome display selection even if UAG codons were present in their sequences. Furthermore, after the evaluation of the incorporation of a photo-cross-linkable non-canonical amino acid, namely *p*-benzoyl-l-phenylalanine (*p*Bzo-Phe), into the UAG positions in peptide coding sequences, the optimal conditions were determined to promote the photo-cross-linking reactions to form covalent bonds between *p*Bzo-Phe-incorporated peptide epitopes and monoclonal antibodies. Finally, we performed and assessed a ribosome display selection of submicromolar low-affinity peptides in combination with genetic code expansion using *p*Bzo-Phe-tRNA^UAG^ and photo-irradiation.

## 2. Results and Discussion

### 2.1. A Scheme of Ribosome Display Selection for Identifying Affinity Peptides

**Figure 1:**
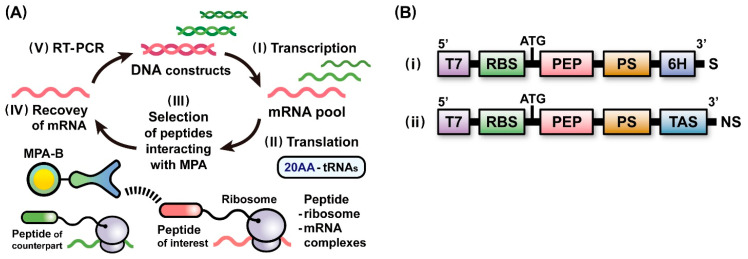
(**A**) Ribosome display selection for identifying the peptide of interest. (I) In vitro transcription of DNA constructs for generating an mRNA pool that encodes various peptides. (II) In vitro translation of the mRNA pool in the presence of 20 canonical amino acid-tRNA types (AA-tRNAs) for forming peptide-ribosome-mRNA complexes that can link the peptides as phenotypes and the corresponding mRNAs as genotypes. (III) In vitro selection of ribosomal complexes that display peptides interacting with monoclonal anti-peptide antibodies immobilized on beads (MPA-B). (IV) Recovery of mRNAs by disassembling the selected ribosomal complexes. (V) Reverse transcription (RT)-PCR for synthesis of DNAs used to perform the next round of selection or to identify peptides. (**B**) Linear DNA constructs for generating mRNAs used to synthesize various peptide-fused proteins (i) or to form peptide-ribosome-mRNA complexes (ii). T7 = T7 promoter for transcription, RBS = ribosome-binding site for translation, ATG = start codon, PEP = coding sequences for peptides (FLAG, V5, TAG-FLAG, TAG-FLAG(K3A), or TAG-FLAG(Y2A)) inserted between two different *Sfi*I restriction sites, TAG = amber stop codon that is converted into UAG through transcription, PS = coding sequence for a protein spacer, 6H = coding sequence for a hexa-histidine tag, S = other stop codons, TAS = translation arrest sequence, and NS = no stop codon.

Prior to the development of ribosome display techniques in combination with genetic code expansion, we aimed to construct a scheme of ribosome display selection to identify peptide epitopes specifically binding to monoclonal antibodies. As shown in Figure 1A, the scheme was designed to provide standards for assessing the processes involved in ribosome display selections of affinity peptides and to be used as a platform for ribosome display techniques combined with genetic code expansions. We first constructed two linear DNAs, DNA(T7-RBS-PEP-PS-6H) (Figure 1B(i)) and DNA(T7-RBS-PEP-PS-TAS) (Figure 1B(ii)), which were to be transcribed in vitro using T7 RNA polymerase. The resultant mRNAs were individually used for protein expression with an *E. coli* cell-free protein synthesis system and the construction of mRNA pools that encoded the peptides of interest and their counterparts (Figure 1A(I)). In the in vitro translation of mRNAs (Figure 1A(II)), to bring a nascent peptide out of the ribosome tunnel and display it on the ribosomal complex, a protein spacer (PS) of an appropriate length was essential for fusion at the C-terminus of each peptide. The translation arrest sequence (TAS) derived from *E. coli* binds to the inside of the ribosome tunnel during the peptide elongation reaction [39,40]. Thus, the insertion of TAS into the C-terminus of PS can support an idling of the ribosome on the 3′ terminus of an mRNA. In addition, to achieve the stable formation of peptide-ribosome-mRNA complexes along with the effect of the TAS, stop codons were removed from the mRNA sequences used for ribosome display selection.

The FLAG peptide was known to interact tightly with a monoclonal anti-FLAG peptide antibody (MFA), and the dissociation constant (*K*d) was estimated to be 0.76 nM [41]. To establish the operations in the scheme (Figure 1A) for identifying affinity peptides interacting with monoclonal anti-peptide antibodies immobilized on beads (MPA-B) as partner molecules, we verified the ribosome display selection of the FLAG peptides in the coexistence of the V5 peptides of the counterpart as follows. First, we preliminarily confirmed whether each peptide-fused protein displayed on the ribosome could be expressed in *E. coli* translational machinery. Accordingly, after in vitro translation of mRNA(RBS-FLAG-PS-6H) and mRNA(RBS-V5-PS-6H), the expression levels of the fusion proteins with hexa-histidine tags at their C-termini, FLAG-PS-6H and V5-PS-6H, were assessed with Western blot analysis using a monoclonal anti-hexa-histidine tag antibody conjugated to a peroxidase (MHA-P) (Figure S1). All fusion proteins were fully expressed using the *E. coli* cell-free protein synthesis system, supporting that these peptides were likely to be displayed on ribosomal complexes. Next, according to the scheme (Figure 1A), DNA(T7-RBS-FLAG-PS-TAS) and DNA(T7-RBS-V5-PS-TAS) were transcribed in vitro to synthesize mRNA(RBS-FLAG-PS-TAS) and mRNA(RBS-V5-PS-TAS), respectively (Figure 1A(I)). The two mRNAs were mixed to construct an initial mRNA pool, and the molar ratio of the mRNA of the FLAG peptide to that of the V5 peptide was adjusted to 1:10^5^. The quantity of mRNA encoding the FLAG peptide was almost identical to that of the mRNA encoding one peptide in a pool of mRNAs encoding 100,000 different species of peptides. Through in vitro translation of the mRNA pool, a pool consisting of an extremely small amount of FLAG peptide-ribosome-mRNA complexes and large amounts of V5 peptide-ribosome-mRNA complexes was obtained (Figure 1A(II)). The resultant pool of ternary complexes was screened against monoclonal anti-FLAG peptide antibodies immobilized on beads (MFA-B) to select specific peptide-ribosome-mRNA complexes that interact with MFAs (Figure 1A(III)). The selected ternary complexes were disassembled by lowering the Mg^2+^ concentration to recover the mRNAs (Figure 1A(IV)). Reverse transcription (RT) of the recovered mRNAs was performed to produce cDNAs. Linear DNAs were synthesized through PCR using the cDNAs as templates to initiate the next round of ribosome display selection (Figure 1A(V)).

**Figure 2:**
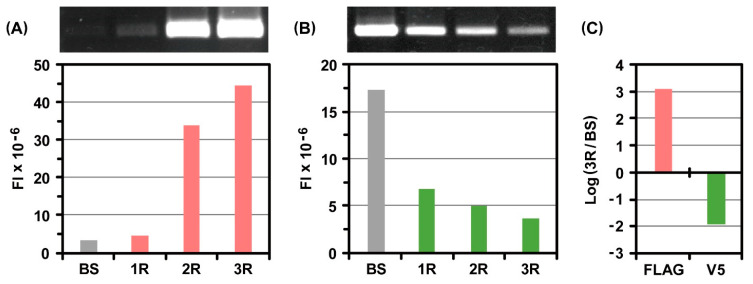
Electrophoretic gel analysis of RT-PCR products derived from the mRNAs that encode (**A**) FLAG peptide or (**B**) V5 peptide after ribosome display selection against monoclonal anti-FLAG peptide antibodies immobilized on beads. FI = fluorescent intensity, BS = before selection, 1R = the first round of selection, 2R = the second round of selection, and 3R = the third round of selection. In the initial mRNA pool, the molar amount of mRNA of the FLAG peptide of interest was 1/10^5^ times that of the V5 peptide as its counterpart. (**C**) Augmentation of FLAG peptide and reduction of V5 peptide after the third round of selection. The RT-PCR products were quantified on the basis of the fluorescent band intensities in the electrophoretic gel image.

After the third round of selection, portions of the initial mRNA pool and mRNAs recovered from each selection were subjected to RT-PCR under the same condition. The resultant RT-PCR products were quantitatively compared with gel electrophoretic analysis. Before selection, the fluorescence band intensity derived from the FLAG peptide was detected at trace levels because of its extremely low concentration (Figure 2A). With an increase in the number of selection rounds, the intensity increased drastically. In contrast, the fluorescence band intensity derived from the V5 peptide gradually decreased (Figure 2B). Based on these results, changes in the abundance ratio of each peptide were estimated before selection and after the third round of selection. The calculated high positive value indicated that the FLAG peptide could be specifically selected as a peptide of interest by repeating ribosome display selection, whereas the calculated negative value represented that the V5 peptide was excluded from the process of selection (Figure 2C).

To prove the suitability of each operation in the scheme (Figure 1A), we evaluated the ribosome display selection of the V5 peptides of interest from among their counterparts. In contrast to the previous selection, the molar ratio of the mRNA of the V5 peptide to that of the FLAG peptide of the counterpart was set to 1:10^5^ in the initial mRNA pool. After the third round of selection against monoclonal anti-V5 peptide antibodies immobilized on beads, electrophoretic gel analysis of the RT-PCR products revealed that the V5 peptide was preferentially enriched with the exclusion of the FLAG peptide (Figure S2). Therefore, these two ribosome display selections were adequately implemented to identify each peptide epitope of interest. The operations in the established scheme (Figure 1A) can be utilized as a platform for development of ribosome display techniques in combination with genetic code expansion.

### 2.2. Incorporation of Genetic Code Expansion into the Ribosome Display Selection

The amber stop codon TAG frequently emerges during the synthesis of DNA sequences comprising tandemly repeated NNK codons. Therefore, the assignment of specified canonical and non-canonical amino acids to amber stop codons using synthetic X-tRNA^UAG^ is expected to suppress immature translational termination in the coding frame and thus maintain the diversity of peptide libraries. To obtain the desired X-tRNA^UAG^ [42,43], a dinucleotide consisting of deoxycytidine and adenosine (pdCpA) was chemically synthesized and modified with the canonical amino acid Tyr or Trp. The resultant Tyr-pdCpA or Trp-pdCpA was individually ligated into synthetic tRNA^UAG^ lacking pCpA at the 3′ terminal, using T4 RNA ligase to produce Tyr-tRNA^UAG^ or Trp-tRNA^UAG^. The backbone of synthetic tRNA^UAG^ was composed of the sequence of *Mycoplasma capricolum* Trp_1_ tRNA to prevent canonical amino acids from being charged at its 3′ terminal by *E. coli* aminoacyl-tRNA synthetases.

To ascertain whether Tyr and Trp could be incorporated into the amber stop codon by synthetic X-tRNA^UAG^ in the *E. coli* translational machinery, we assessed the expression levels of the peptide-fused proteins Tyr-FLAG-PS-6H and Trp-FLAG-PS-6H through Western blot analysis. In the case of in vitro translation of mRNA(RBS-UAG-FLAG-PS-6H) without X-tRNA^UAG^, no proteins were produced because of the action of translation termination factors. However, after in vitro translation in the presence of Tyr-tRNA^UAG^ and Trp-tRNA^UAG^, the two fusion proteins were sufficiently expressed, and their maximum expression levels were observed in the presence of a 2.0 µL of X-tRNA^UAG^ (Figure S3). At the beginning of translation, the sterically bulky amino acids in the nascent peptide stabilize the ribosome to ensure continuous elongation [44]. Thus, the incorporation of Trp before the sequence of FLAG peptide might be an advantage for the efficient expression of the fusion protein compared with that of Tyr.

**Figure 3:**
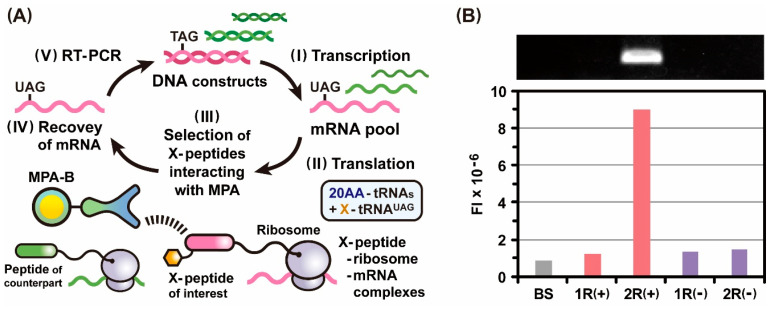
(**A**) Ribosome display selection in combination with genetic code expansion for identifying the X-peptide of interest. (I) In vitro transcription of DNA constructs, including an amber stop codon (TAG) for generating an mRNA pool that encodes various peptides. (II) In vitro translation of the mRNA pool in the presence of 20 types of AA-tRNAs and synthetic X-tRNA^UAG^ to form X-peptide-ribosome-mRNA complexes that can link the X-peptides as phenotypes and the corresponding mRNAs as genotypes. (III) In vitro selection of ribosomal complexes that display X-peptides interacting with monoclonal anti-peptide antibodies immobilized on beads (MPA-B). (IV) Recovery of mRNAs by disassembling the selected ribosomal complexes. (V) Reverse transcription (RT)-PCR for synthesis of DNAs used to perform the next round of selection or to identify X-peptides. (**B**) Electrophoretic gel analysis of RT-PCR products derived from the mRNAs that encode the sequences of UAG-FLAG peptides after the ribosome display selection against MFA-B. FI = fluorescent intensity, BS = before selection, 1R = the first round of selection, and 2R = the second round of selection. In the initial mRNA pool, the molar amount of mRNA of the UAG-FLAG peptide of interest was 1/10^5^ times that of the V5 peptide as its counterpart. The mRNAs were recovered in each round of selection after in vitro translation in the presence (+) or absence (−) of Trp-tRNA^UAG^. The RT-PCR products were quantified on the basis of the fluorescent band intensities in the electrophoretic gel image.

Assuming that an amber stop codon appeared in the coding sequences for the peptides of interest in the course of the construction of DNA libraries, we incorporated a strategy of genetic code expansion using X-tRNA^UAG^ into the ribosome display selection (Figure 3A) and verified whether X-incorporated peptides could be identified in the presence of extremely large amounts of peptides of non-interest as follows. DNA(T7-RBS-TAG-FLAG-PS-TAS) and DNA(T7-RBS-V5-PS-TAS) were transcribed in vitro to generate the corresponding mRNAs (Figure 3A(I)). An initial mRNA pool was constructed by mixing mRNA(RBS-UAG-FLAG-PS-TAS) and mRNA(RBS-V5-PS-TAS). The molar ratio of the mRNA of the UAG-FLAG peptide to that of the V5 peptide was set to 1:10^5^. Thus, after in vitro translation of the mRNA pool in the presence of Trp-tRNA^UAG^, the amount of Trp-FLAG peptide-ribosome-mRNA complexes was expected to be equal to or lower than 1/10^5^ times that of the V5 peptide-ribosome-mRNA complexes (Figure 3A(II)). The pool consisting of ribosomal complexes was screened against MFA-B to select specific peptide-ribosome-mRNA complexes through affinity interactions with MFAs (Figure 3A(III)). The selected ribosomal complexes were dissociated to recover their mRNAs (Figure 3A(IV)), and RT-PCR was conducted by using the recovered mRNAs to synthesize the linear DNAs used for the next round of ribosome display selection (Figure 3A(V)).

After the second round of selection, we carried out RT-PCR for the initial mRNA pool and the mRNAs recovered from each selection. Gel electrophoretic analysis of the RT-PCR products showed that the intensity of the fluorescence band derived from the Trp-FLAG peptide drastically increased in the second round of selection (Figure 3B). In contrast, no significant increase in the quantity of Trp-FLAG peptides was observed in the repeated selection without genetic code expansion using Trp-tRNA^UAG^ (Figure 3B). These findings clearly indicate that Trp-FLAG peptide-ribosome-mRNA complexes were formed via site-specific incorporation of Trp at the position dictated by the UAG codon and were specifically selected through affinity interactions with MFAs. Considering the codon misrecognition by synthetic X-tRNA^UAG^, the rate was expected to be quite low due to the tight G-C pair at the wobble position, even if undesirable peptides produced by the codon misrecognition were to be excluded in the next selection round because they were not encoded by any of the retrieved mRNAs. Taken together, the use of X-tRNA^UAG^ is suitable for incorporating a specified amino acid in response to the UAG codons, which can conserve the diversity of peptides subjected to ribosome display selection. The ribosome display technique with genetic code expansion using X-tRNA^UAG^ could be a powerful approach to effectively identifying peptide epitopes of monoclonal antibodies [36,37,38].

### 2.3. Evaluation of the Photo-Cross-Linking Reactions of pBzo-Phe-Incorporated Peptides

Photo-cross-linkable compounds can be activated by photo-irradiation at specific wavelengths to readily form covalent bonds with adjacent molecules [45,46,47,48,49]. Recently, the insertion of photo-cross-linkable amino acids into protein structures has been applied to analyze protein–protein interactions [34,35]. In Section 2.2, we demonstrated that in vitro translation of mRNA(RBS-UAG-FLAG-PS-TAS) coupled with Trp-tRNA^UAG^ led to the formation of Trp-FLAG peptide-ribosome-mRNA complexes used for ribosome display selection. Thus, through the application of genetic code expansion using X-tRNA^UAG^, a photo-cross-linkable amino acid can be incorporated into the desired site in the peptide sequence displayed on the ribosomal complex. Moreover, the resultant photo-cross-linkable ribosomal complexes are expected to be utilized to select low-affinity peptides that are covalently bound to partner molecules after photo-irradiation. In this study, *p*Bzo-Phe was selected as the photo-cross-linkable amino acid because the *p*-benzoyl phenyl unit can generate a benzoyl radical via photo-irradiation to covalently bind to an adjacent molecule in an aqueous solution [49]. In addition, if a covalent bond is not formed after photo-irradiation, then the moderate reactivity of the stable radical allows it to return to the original *p*-benzoyl phenyl unit and reactivation by photo-irradiation. Unlike the trifluoromethyldiazirine and azidophenyl units commonly used for photo-cross-linking reactions [45,46,47,48], these properties are suitable for efficient photo-cross-linking reactions between low-affinity peptides and partner molecules while minimizing non-specific covalent bond formation.

**Figure 4:**
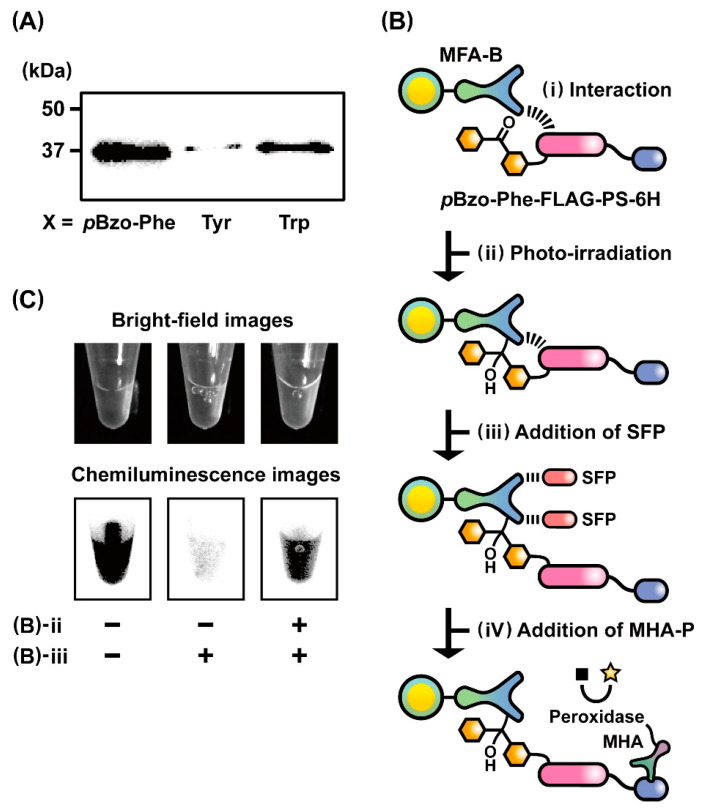
(**A**) Western blot analysis of the expression levels of X-FLAG peptides (X = *p*Bzo-Phe, Tyr, and Trp), depending on the type of synthetic X-tRNA^UAG^. To generate X-FLAG peptides fused with a protein spacer (PS) and a hexa-histidine tag (6H), namely *p*Bzo-Phe-FLAG-PS-6H, Tyr-FLAG-PS-6H, and Trp-FLAG-PS-6H, in vitro translations of the mRNA encoding the sequence of UAG-FLAG-PS-6H were performed in the presence of *p*Bzo-Phe-tRNA^UAG^, Tyr-tRNA^UAG^, and Trp-tRNA^UAG^, respectively. (**B**) Schematic representation for analyzing photo-cross-linking reaction between *p*Bzo-Phe-FLAG peptide-fused protein (*p*Bzo-Phe-FLAG-PS-6H) and MFA-B. (i) Interaction of *p*Bzo-Phe-FLAG-PS-6H with MFA. (ii) Photo-irradiation for activating *p*Bzo-Phe unit and inducing photo-cross-linking reaction. (iii) Addition of synthetic FLAG peptides (SFPs) for eluting *p*Bzo-Phe-FLAG-PS-6H that did not bind to MFA-B via a covalent bond. (iv) Addition of monoclonal anti-hexa-histidine tag antibody conjugated with a peroxidase (MHA-P) for detecting the fusion proteins bound covalently to MFA-B by chemiluminescence. (**C**) Bright-field and chemiluminescence images of MFA-B with or without photo-irradiation and addition of SFPs.

To confirm whether the *p*Bzo-Phe unit could be introduced into the position of the amber stop codon in the designated peptide sequence, the expression level of a peptide-fused protein, *p*Bzo-Phe-FLAG-PS-6H, was assessed after in vitro translation of mRNA(RBS-UAG-FLAG-PS-6H) in the presence of *p*Bzo-Phe-tRNA^UAG^. Western blot analysis revealed that the entire sequence of *p*Bzo-Phe-FLAG-PS-6H was expressed in the *E. coli* translational machinery (Figure 4A). Notably, its expression level was much higher than those of Tyr-FLAG-PS-6H and Trp-FLAG-PS-6H, which were produced by in vitro translation of mRNA(RBS-UAG-FLAG-PS-6H) in the presence of Tyr-tRNA^UAG^ and Trp-tRNA^UAG^, respectively. This trend in the expression levels may reflect that the peptide elongation reaction depends on the size of the sterically bulky amino acids at the beginning of translation [44].

To verify whether *p*Bzo-Phe-incorporated FLAG peptides could covalently bind to MFAs through photo-irradiation, we performed photo-cross-linking reactions between the *p*Bzo-Phe-FLAG peptide-fused proteins, *p*Bzo-Phe-FLAG-PS-6H, and MFA-B according to the protocol presented in Figure 4B. The translation solution of *p*Bzo-Phe-FLAG-PS-6H was gently mixed with MFA-B to promote interactions between the fusion protein and MFA (Figure 4B(i)). Irradiation with light at a wavelength of 365 nm activated the *p*Bzo-Phe unit and induced a photo-cross-linking reaction of the fusion protein with MFA (Figure 4B(ii)). Synthetic FLAG peptides (SFPs) were added to elute the fusion proteins that did not bind to MFAs via covalent bonds (Figure 4B(iii)). Finally, after the addition of MHA-P to the solution containing MFA-B, the fusion protein covalently bound to MFA was specifically detected by measuring the chemiluminescence from the reaction of MHA-P with a light-emitting reagent (Figure 4B(iv)). As a positive control, chemiluminescence was observed under the condition without photo-irradiation and an SFP addition. The result showed that the fusion proteins were presented on the surface of MFA-B via non-covalent affinity interactions with MFAs (Figure 4C, left). In contrast, strong light emission was not observed under the condition with an SFP addition in the absence of photo-irradiation, revealing that most of the fusion proteins were eluted through competitive binding of SFPs to MFAs (Figure 4C, middle). As expected, substantial chemiluminescence was detected after photo-irradiation, even when SFPs were added to the solution of MFA-B, indicating the formation of covalent bonds between the fusion proteins and MFAs (Figure 4C, right). Therefore, chemiluminescence image analysis supports the possibility of ribosome display selection of *p*Bzo-Phe-peptide-fused proteins covalently bound to MFAs through photo-irradiation.

### 2.4. Photo-Cross-Linkable Ribosome Display Selection for Identifying Low-Affinity Peptides

The replacement of Lys3 or Tyr2 with Ala in the FLAG peptide sequence (DYKDDDDK) drastically lowered its affinity for MFA [50]. Through a calculation based on the binding constants, the *K*d values of the FLAG variant peptides FLAG(K3A) and FLAG(Y2A) were estimated to be approximately 90 and 230 nM, respectively. In particular, the affinity of the FLAG(Y2A) peptide for MFA was 300-fold lower than that of the original FLAG peptide. Here, to confirm whether the *p*Bzo-Phe unit could be incorporated into the position of the amber stop codon before the sequences of FLAG variant peptides, mRNA(RBS-UAG-FLAG-PS-6H), mRNA(RBS-UAG-FLAG(K3A)-PS-6H), and mRNA(RBS-UAG-FLAG(Y2A)-PS-6H) were translated in vitro in the presence of *p*Bzo-Phe-tRNA^UAG^, and the expression levels of the fusion proteins *p*Bzo-Phe-FLAG-PS-6H, *p*Bzo-Phe-FLAG(K3A)-PS-6H, and *p*Bzo-Phe-FLAG(Y2A)-PS-6H were compared with Western blot analysis (Figure S4). The full-length sequences of *p*Bzo-Phe-FLAG(K3A)-PS-6H and *p*Bzo-Phe-FLAG(Y2A)-PS-6H were expressed. However, their expression levels were lower than that of *p*Bzo-Phe-FLAG-PS-6H. The decrease associated with alanine mutations might be attributed to the instability of the ribosome through replacing the sterically bulky amino acids Lys and Tyr with a smaller amino acid Ala in the FLAG peptide sequence. Nonetheless, the observed expression of these fusion proteins could lead to the formation of *p*Bzo-Phe-FLAG variant peptide-ribosome-mRNA complexes using the *E. coli* cell-free protein synthesis system.

To develop a photo-cross-linkable ribosome display technique for identifying low-affinity peptides that interact specifically with partner molecules, we performed a ribosome display selection of *p*Bzo-Phe-FLAG(Y2A) peptides after photo-irradiation and validated the operations as follows. Based on the scheme (Figure 3A), DNA(T7-RBS-TAG-FLAG(Y2A)-PS-TAS) and DNA(T7-RBS-V5-PS-TAS) were transcribed in vitro to generate mRNA(RBS-UAG-FLAG(Y2A)-PS-TAS) and mRNA(RBS-V5-PS-TAS), respectively (Figure 3A(I)). The initial mRNA pool was designed to contain mRNA(RBS-UAG-FLAG(Y2A)-PS-TAS) and mRNA(RBS-V5-PS-TAS) at a molar ratio of 1:10^5^. Therefore, after in vitro translation of the mRNA pool in the presence of *p*Bzo-Phe-tRNA^UAG^, the quantity of the *p*Bzo-Phe-FLAG(Y2A) peptide-ribosome-mRNA complex of interest would be lower than 1/10^5^ times that of the V5 peptide-ribosome-mRNA complex as its counterpart (Figure 3A(II)). The pool of ribosomal complexes was mixed with MFA-B to promote interactions between the *p*Bzo-Phe-FLAG(Y2A) peptides and MFAs. The beads were irradiated with a light having a wavelength of 365 nm for inducing photo-cross-linking reactions between the *p*Bzo-Phe units and MFAs (Figure 3A(III)). An excessive amount of SFPs was added to the beads to competitively elute ribosomal complexes that did not form covalent bonds with MFAs. The *p*Bzo-Phe-FLAG(Y2A) peptide-ribosome-mRNA complexes covalently bound to MFAs were selected and disassembled by decreasing the Mg^2+^ levels to recover their mRNAs (Figure 3A(IV)). RT-PCR of the recovered mRNAs was performed to produce linear DNAs suitable for the next round of ribosome display selection (Figure 3A(V)).

**Figure 5:**
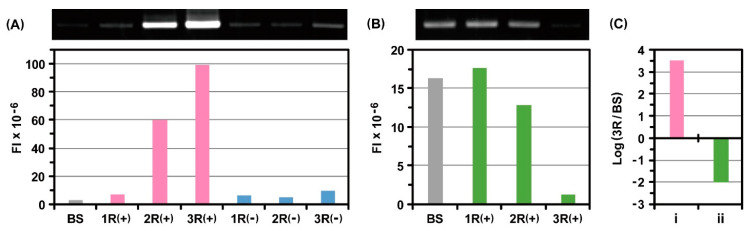
Electrophoretic gel analysis of RT-PCR products derived from the mRNAs that encode (**A**) UAG-FLAG(Y2A) peptide or (**B**) V5 peptide after ribosome display selection against MFA-B. FI = fluorescent intensity, BS = before selection, 1R = the first round of selection, 2R = the second round of selection, and 3R = the third round of selection. In the initial mRNA pool, the molar amount of mRNA of the UAG-FLAG(Y2A) peptide of interest was 1/10^5^ times that of the V5 peptide as its counterpart. Then, *p*Bzo-Phe was inserted into the position of the amber stop codon through in vitro translation in the presence of *p*Bzo-Phe-tRNA^UAG^, leading to the formation of *p*Bzo-Phe-FLAG(Y2A) peptide-ribosome-mRNA complexes. The mRNAs were recovered in each round of selection after photo-irradiation (+) or non-irradiation (−), followed by the addition of synthetic FLAG peptides. (**C**) Augmentation of *p*Bzo-Phe-FLAG(Y2A) peptide (i) and reduction of V5 peptide (ii) after the third round of ribosome display selection. The RT-PCR products were quantified on the basis of the fluorescent band intensities in the electrophoretic gel image.

After the third round of selection, the RT-PCR products of the initial mRNA pool and the mRNAs obtained in each selection were analyzed using gel electrophoresis. As the selections with photo-irradiation were repeated, the fluorescence band intensity derived from the *p*Bzo-Phe-FLAG(Y2A) peptide increased dramatically (Figure 5A). In contrast, the fluorescence band intensity of the V5 peptide decreased after repeated selection (Figure 5B). Additionally, the abundance ratios of the two peptides after the final selection were compared by calculating the changes in their fluorescence band intensities (Figure 5C). The positive value of the *p*Bzo-Phe-FLAG(Y2A) peptide represented an increase in its quantity, whereas the negative value of the V5 peptide indicated a decrease. These data evidently signify that the *p*Bzo-Phe-FLAG(Y2A) peptide of interest was dominantly selected, and the V5 peptide as its counterpart was excluded with increasing selection rounds. Contrastingly, in the repeat of selections without photo-irradiation, an increase in the fluorescence band intensity of the *p*Bzo-Phe-FLAG(Y2A) peptide was not observed (Figure 5A). This phenomenon strongly suggests that augmentation of the *p*Bzo-Phe-FLAG(Y2A) peptide occurred through the specific selection of the *p*Bzo-Phe-FLAG(Y2A) peptide-ribosome-mRNA complexes bound covalently to MFAs via photo-irradiation. Thus, these results demonstrate that the ribosome display technique in combination with genetic code expansion using *p*Bzo-Phe-tRNA^UAG^ allows the specific identification of peptides of interest through photo-cross-linking reactions, even if they have submicromolar affinity to partner molecules. In the phage and mRNA display techniques, peptides as phenotypes displayed on the coat proteins of bacteriophages and the termini of mRNAs cannot be separated from the corresponding genes as genotypes [22,23,24,25,26,27]. On the other hand, the peptide-ribosome-mRNA complexes used in the ribosome display technique can be properly disassembled under the mild condition that the Mg^+^ levels are lowered in the solution [18,19,20,21]. Therefore, the unique characteristics of the phenotype-genotype complex enabled the efficient recovery of mRNAs encoding affinity peptides, even if they were bound covalently to partner molecules. In the use of the cell-free protein synthesis system with translation release factors, even the inclusion of X-tRNA^UAG^ would not completely suppress the translation termination through in-frame UAG codons. Hence, the removal of the factors from a cell-free mixture will lead to further improving the selection efficiency. Thus, photo-cross-linkable ribosome display selection could be a powerful method for exploring specific peptides with a wide range of affinities to desired partner molecules, including antibodies [15,36,37,38].

## 3. Materials and Methods

### 3.1. Construction of Plasmid and Linear DNAs

The basic plasmid, pDNA(RBS-2*Sfi*I-PS-TAS), was constructed based on the sequence of p(*Sfi*I2-Ps) previously reported [20]. The plasmid contained a ribosome-binding site, two different sites of *Sfi*I, a coding sequence for a protein spacer consisting of 278 amino acids, and a translation arrest sequence derived from the *E. coli* SecM protein [39,40]. The oligonucleotides shown in Table S1 were chemically synthesized, and their 5′-termini were phosphorylated using T4 Polynucleotide Kinase (TAKARA, Kusatsu-shi, Japan). Through hybridization of the corresponding phosphorylated oligonucleotides, double-strand DNA fragments encoding a series of peptides (PEP: FLAG, V5, TAG-FLAG, TAG-FLAG(K3A), and TAG-FLAG(Y2A)) were prepared. These fragments were individually inserted between the *Sfi*I sites of pDNA(RBS-2*Sfi*I-PS-TAS) to produce pDNA(RBS-PEP-PS-TAS). Next, the linear DNA, DNA(T7-RBS-PEP-PS-6H), as represented in Figure 1B(i), was constructed using PCR with PrimeSTAR GXL DNA polymerase (TAKARA), pDNA(RBS-PEP-PS-TAS) as a template, and the primer pairs (fp-T7 to introduce the T7 promoter sequence TTAATACGACTCACTATAGAAAAGTCGACAATAATTTTGTTTAACTT and rp-6H to introduce the sequence encoding a hexa-histidine tag TCATTAATGATGGTGGTGGTGGTGCTGGATCCCATCGATAGC). Similarly, the linear DNA, DNA(T7-RBS-PEP-PS-TAS), as represented in Figure 1B(ii), was synthesized via PCR with PrimeSTAR GXL DNA polymerase (TAKARA), pDNA(RBS-PEP-PS-TAS) as a template, and the primer pairs (fp-T7 and rp-NS: AAACAGCTATGACCATGATTA). The PCR products were purified using a QIAquick PCR purification kit (Qiagen, Hilden, Germany) and used to synthesize mRNAs suitable for in vitro translation or formation of peptide-ribosome-mRNA complexes.

### 3.2. Western Blot Analysis of Protein Expression

Using RiboMAX Large-Scale RNA Production Systems (Promega, Madison, WI, USA), DNA(T7-RBS-PEP-PS-6H) (Figure 1B(i)) was transcribed in vitro to synthesize mRNA(RBS-PEP-PS-6H). After purification using an RNeasy Mini Kit (Qiagen), the resulting mRNAs (30 pmol) were independently translated in vitro at 37 °C for 3 h in 25 µL of a solution that contained a cell-free protein synthesis reagent (PURE*frex*; GeneFrontier, Kashiwa-shi, Japan) and RNasin Plus Ribonuclease Inhibitor (Promega) in the absence or presence of synthetic X-tRNA^UAG^ (X = Tyr, Trp, or *p*Bzo-Phe; Protein *Express*, Kisarazu-shi, Japan). Translation solutions of the peptide-fused proteins, PEP-PS-6H and X-PEP-PS-6H, were fractionated with SDS-PAGE (12% polyacrylamide gels) in a Tris-glycine buffer. The fusion proteins were subsequently transferred to Hybond-P PVDF membrane (Amersham, Amersham, UK) and allowed to react with a monoclonal anti-hexa-histidine tag antibody conjugated to a peroxidase (Anti 6×Histidine, Monoclonal Antibody, Peroxidase Conjugated, FUJIFILM Wako Pure Chemical, Osaka, Japan) in a solution containing 5% Block Ace Powder (KAC, Kyoto, Japan). The protein bands were visualized using SuperSignal West Pico Plus Chemiluminescent Substrate (ThermoFisher Scientific, Waltham, MA, USA) and then detected using ImageQuant LAS 4000 mini (GE Healthcare, Chicago, IL, USA).

### 3.3. Ribosome Display Selections with or without Genetic Code Expansion

In vitro transcription of DNA(T7-RBS-PEP-PS-TAS) (Figure 1B(ii)) was conducted using RiboMAX Large-Scale RNA Production Systems (Promega) to synthesize mRNA(RBS-PEP-PS-TAS). The resulting mRNAs were purified using an RNeasy Mini Kit (Qiagen). As described in Section 2, the initial RNA pools were prepared individually for each ribosome display selection. In vitro translations were separately carried out using PURE*frex* (GeneFrontier) at 37 °C for 15 min in 25 µL of a solution containing 2.4 pmol of the mRNA pool and 1 µL of RNasin Plus Ribonuclease Inhibitor (Promega) with or without synthetic Trp-tRNA^UAG^ (2 µL). Translation solutions containing peptide-displayed ribosomal complexes were mixed with 150 µL of Buffer S (100 mM Tris-AcO (pH 7.5), 300 mM KCl, 100 mM Mg(AcO)_2_, and 2% Tween 20), 65 µL of dH_2_O, and 60 µL of bovine serum albumin (ThermoFisher Scientific). The resulting solutions were added to 15 µL of MFA-B (ANTI-FLAG M2 Affinity Gel, Merck, Darmstadt, Germany) or monoclonal anti-V5 peptide antibodies immobilized on beads which consisted of Anti-V5-tag mAb-Biotin (MBL, Tokyo, Japan) and High-Capacity NeutrAvidin Agarose (ThermoFisher Scientific). These beads were preliminary incubated in Buffer W (50 mM Tris-AcO (pH 7.5), 150 mM KCl, 50 mM Mg(AcO)_2_, and 1% Tween 20) containing 2% bovine serum albumin (ThermoFisher Scientific). After gentle rotation of the bead mixtures at 4 °C for 20 min and washing several times with 500 µL of Buffer W, approximately 100 µL of Buffer E (50 mM Tris-AcO (pH 7.5), 150 mM KCl, 80 mM ethylenediamine-*N*,*N*,*N*′,*N*′-tetraacetic acid) was added to the beads. The resulting solutions were gently shaken for 30 min to dissociate the ribosomal complexes bound to the bead surfaces. The supernatants were carefully collected, and the mRNAs obtained from the selected ribosomal complexes were purified using an RNeasy Mini Kit (Qiagen). The recovered mRNAs were reverse transcribed at 50 °C for 60 min in a reaction solution containing 4.5 µL of PrimeSTAR Reverse Transcriptase (TaKaRa), 5 µL of dNTP Mix (Promega), 1 µL of RNasin Plus Ribonuclease Inhibitor (Promega), and 25 pmol of the reverse primer rp-NS. The generated cDNAs were subjected to PCR to construct linear DNAs that were used to initiate the next round of ribosome display selection.

### 3.4. RT-PCR and Electrophoretic Gel Analysis after Ribosome Display Selections

Parts of the initial mRNA pools or the mRNAs recovered from each ribosome display selection were reverse transcribed under the same conditions at 50 °C for 60 min in a reaction mixture that contained 2 µL of PrimeSTAR Reverse Transcriptase (TaKaRa), 2 µL of dNTP Mix (Promega), 0.5 µL of RNasin Plus Ribonuclease Inhibitor (Promega), and 10 pmol of rp-NS. Subsequently, the first PCRs were performed in solutions containing PrimeSTAR GXL DNA polymerase (TAKARA), with the synthesized cDNAs as templates and the primer pairs fp-T7 and rp-NS. After purification of the PCR products using a QIAquick PCR purification kit (Qiagen), the secondary PCRs were conducted in solutions containing PrimeSTAR GXL DNA polymerase (TaKaRa), with the first PCR products (0.5 ng) as templates, the oligonucleotides (Table S1) that recognize the coding sequences for a series of peptides (FLAG-s, V5-s, TAG-FLAG-s, TAG-FLAG(K3A)-s, and TAG-FLAG(Y2A)-s), and the reverse primer (rp-PS: AGTCGTTCTTCTCGTACAC). The second PCR products were separated on 1% agarose gels, and the bands were stained for 30 min with ethidium bromide in TAE buffer. The fluorescent band intensities of the PCR products from ribosome display selection and photo-cross-linkable ribosome selection were quantitatively analyzed using an ImageQuant LAS 4000 mini (GE Healthcare) and GelDoc Go Imaging System (BIO-RAD, Hercules, CA, USA), respectively.

### 3.5. Chemiluminescent Image Analysis of Photo-Cross-Linking Reactions

MFA-B (ANTI-FLAG M2 Affinity Gel; Merck) was suspended in Buffer W containing 2% bovine serum albumin (ThermoFisher Scientific). After rotating the beads at 4 °C for 60 min and washing with Buffer W, the beads (15 µL) were individually mixed with a solution (150 µL) containing a photo-cross-linkable peptide-fused protein, *p*Bzo-Phe-FLAG-PS-6H, which was synthesized through in vitro translation of mRNA(RBS-UAG-FLAG-PS-6H) with PURE*frex* (GeneFrontier) and synthetic *p*Bzo-Phe-tRNA^UAG^. The mixtures were softly rotated at 4 °C for 20 min and washed several times with 500 µL of Buffer W. To promote a photo-cross-linking reaction of *p*Bzo-Phe-FLAG-PS-6H with the MFA, the *p*-benzoyl phenyl unit was activated through photo-irradiation with a wavelength of 365 nm for 30 min using LED365-SPT/L (OptoCode, Tokyo, Japan). After several washes with 500 µL of Buffer W and the addition of a solution (100 µL, 200 µg/mL) of synthetic FLAG peptide (Merck), the beads were gently rotated at 4 °C for 30 min. To remove non-specifically bound proteins, the washing of the beads and the addition of synthetic FLAG peptide were carried out twice. Then, the beads were washed three times with 500 µL of Buffer W and added to a diluted solution containing a monoclonal anti-hexa-histidine tag antibody conjugated to a peroxidase (Anti 6×Histidine, Monoclonal Antibody, Peroxidase Conjugated, FUJIFILM Wako Pure Chemical). After incubation at 4 °C for 20 min and several washes with 500 µL of Buffer W, chemiluminescence reactions were facilitated by adding 50 µL of SuperSignal West Pico Plus Chemiluminescent Substrate (ThermoFisher Scientific). Bright-field and chemiluminescent images of the beads were directly acquired using an ImageQuant LAS 4000 mini (GE Healthcare).

### 3.6. Photo-Cross-Linkable Ribosome Display Selection with Genetic Code Expansion

The initial mRNA pools (2.4 pmol) were translated at 37 °C for 15 min in 25 µL of a solution containing PURE*frex* (GeneFrontier) and an RNasin Plus Ribonuclease Inhibitor (Promega) with 2 µL of synthetic *p*Bzo-Phe-tRNA^UAG^. The translation solutions were diluted with 150 µL of Buffer S, 65 µL of dH_2_O, and 60 µL of bovine serum albumin (ThermoFisher Scientific) and then mixed with 15 µL of MFA-B (ANTI-FLAG M2 Affinity Gel, Merck). After gently rotating the beads at 4 °C for 20 min and washing several times with Buffer W, they were irradiated with a 365 nm light for 30 min using LED365-SPT/L (OptoCode). Next, the beads were washed thrice with 500 µL of Buffer W, mixed with a solution (100 µL, 200 µg/mL) of synthetic FLAG peptide (Merck), and subsequently rotated at 4 °C for 30 min. The washing of the beads and mixing of them with synthetic FLAG peptides were conducted twice. The resulting beads were washed several times with 500 µL of Buffer W and added to approximately 100 µL of Buffer E. After gentle shaking to dissociate the ribosomal complexes bound to the bead surfaces, the mRNAs in the supernatants were purified using an RNeasy Mini Kit (Qiagen). The obtained mRNAs were reverse transcribed using PrimeSTAR Reverse Transcriptase (TaKaRa) and rp-NS. The generated cDNAs were used as templates for PCR to synthesize linear DNAs, which were used in the next round of ribosome display selection.

## 4. Conclusions

When selecting affinity peptides from peptide libraries using cell-free molecular display techniques, the appearance of amber stop codons causes translational termination of mRNAs encoding combinatorial peptides, resulting in a reduction in the number of affinity peptide candidates. Moreover, during selections under improper conditions, the amplification of non-specific peptides frequently occurs, hindering the identification of desirable low-affinity peptides. In this study, to maintain the diversity of peptides and identify affinity peptides that interact specifically with partner molecules, we developed the ribosome display techniques combined with genetic code expansion using synthetic X-tRNA^UAG^ and photo-cross-linking reactions. Initially, we designed a scheme for ribosome display selections of the FLAG and V5 peptides against monoclonal anti-peptide antibodies as partner molecules. The analysis after repeating the selections indicated that the proposed operations were able to identify the peptide epitopes of interest, and the established scheme provided a platform of ribosome display techniques coupled with genetic code expansion. Next, based on the observed high efficiency of in vitro translation with synthetic Trp-tRNA^UAG^, we incorporated genetic code expansion using Trp-tRNA^UAG^ into the scheme. The quantification of mRNAs obtained from each ribosome display selection revealed that Trp was incorporated into the position dictated by the amber stop codon in the peptide sequence, leading to specific identification of the Trp-FLAG peptide epitopes. Furthermore, the highest efficiency of cell-free protein expression was observed when using synthetic *p*Bzo-Phe-tRNA^UAG^ in comparison with those using Trp-tRNA^UAG^ and Tyr-tRNA^UAG^. The formation of a covalent bond between the *p*Bzo-Phe-integrated FLAG peptide and MFA was confirmed after photo-irradiation. According to the findings, we finally performed ribosome display selection of low-affinity FLAG variant peptides in combination with genetic code expansion using *p*Bzo-Phe-tRNA^UAG^ and photo-irradiation under optimal conditions. The results demonstrated that the *p*Bzo-Phe-FLAG(Y2A) peptide-ribosome-mRNA complexes could be photo-chemically activated to bind covalently to MFAs and that ribosome display selection enabled the identification of submicromolar low-affinity peptide epitopes. Thus, through further enhancement of the peptide diversity, the photo-cross-linkable ribosome display technique could contribute to efficient identification of undiscovered affinity peptides, thereby promoting versatile peptide-based research in the fields of biotechnology and drug discovery [15,16,17,36,37,38].

## Data Availability

Data are contained within the article or Supplementary Materials.

## Supplementary Materials

The following supporting information can be downloaded at https://www.mdpi.com/article/10.3390/ijms242115661/s1.
