# Supplementary material for: Genetic Code Expansion and a Photo-Cross-Linking Reaction Facilitate Ribosome Display Selections for Identifying a Wide Range of Affinity Peptides
Source: Int J Mol Sci. 2023 Oct 27;24(21):15661. doi: 10.3390/ijms242115661 (PMC10650079; doi:10.3390/ijms242115661)
Supplement: Supplementary file 1 [file ijms-24-15661-s001.zip › ijms-2618638-supplementary.pdf]

Supplementary Materials:

Genetic Code Expansion and a Photo-Cross-Linking Reaction Facilitate Ribosome Display Selections for Identifying a Wide Range of Affinity Peptides

Takuto Furuhashi,[1,2] Kensaku Sakamoto,[3,4] and Akira Wada [1,2,3,*]

[1] Graduate School of Medical Life Science, Yokohama City University, 1-7-29 Suehiro-cho, Tsurumi-ku, Yokohama, Kanagawa 230-0045, Japan

[2] Laboratory for Advanced Biomolecular Engineering, RIKEN Center for Biosystems Dynamics Research, 1-7-22 Suehiro-cho, Tsurumi-ku, Yokohama, Kanagawa 230-0045, Japan

[3] Laboratory for Nonnatural Amino Acid Technology, RIKEN Center for Biosystems Dynamics Research, 1-7-22 Suehiro-cho, Tsurumi-ku, Yokohama, Kanagawa 230-0045, Japan

[4] Department of Drug Target Protein Research, School of Medicine, Shinshu University, 3-1-1 Asahi, Matsumoto, Nagano 390-8621, Japan

* E-mail: awada@riken.jp (A.W.)

Contents:

Table S1 -- pS2

Figure S1 – Figure S4 -- pS3 – pS4

Table S1. Oligonucleotides for constructing double strand DNA fragments that encode various peptides

Name	Sequence
FLAG-s	AGGCCGACTACAAGGACGATGACGACAAGGGCCAGCT
FLAG-as	TGGCCCTTGTCGTCATCGTCCTTGTAGTCGGCCTGCA
V5-s	AGGCCGGTAAGCCAATCCCAAACCCGCTCCTCGGTCTGGATTCTACTGGCCAGCT
V5-as	TGGCCAGTAGAATCCAGACCGAGGAGCGGGTTTGGGATTGGCTTACCGGCCTGCA
TAG-FLAG-s	AGGCCTAGGACTACAAGGACGATGACGACAAGGGCCAGCT
TAG-FLAG-as	TGGCCCTTGTCGTCATCGTCCTTGTAGTCCTAGGCCTGCA
TAG-FLAG(K3A)-s	AGGCCTAGGACTACGCTGACGATGACGACAAGGGCCAGCT
TAG-FLAG(K3A)-as	TGGCCCTTGTCGTCATCGTCAGCGTAGTCCTAGGCCTGCA
TAG-FLAG(Y2A)-s	AGGCCTAGGACGCTAAGGACGATGACGACAAGGGCCAGCT
TAG-FLAG(Y2A)-as	TGGCCCTTGTCGTCATCGTCCTTAGCGTCCTAGGCCTGCA

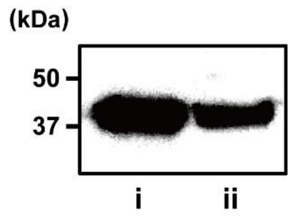

Figure S1. Western blot analysis of the expression levels of FLAG peptide and V5 peptide fused with a protein spacer (PS) and a hexa-histidine tag (6H), FLAG-PS-6H (i) and V5-PS-6H (ii).

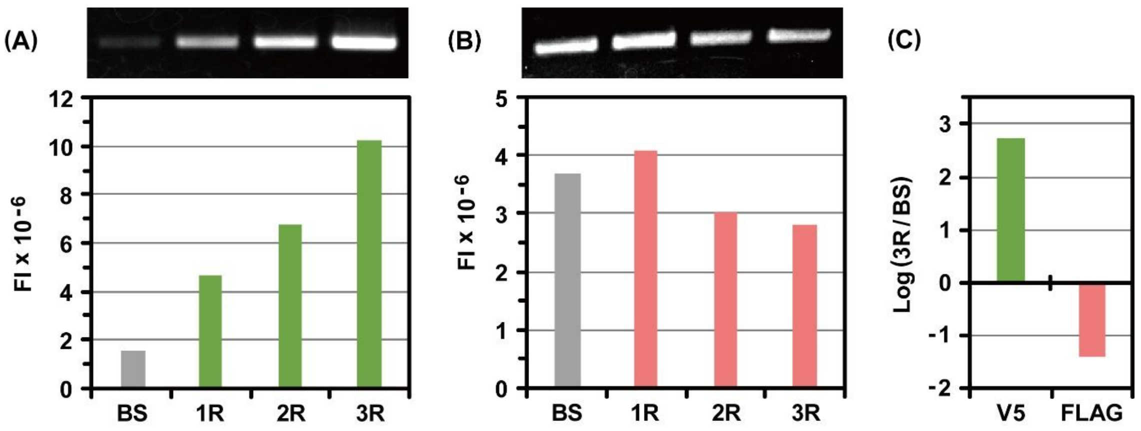

Figure S2. Electrophoretic gel analysis of RT-PCR products derived from the mRNAs that encode (A) V5 peptide or (B) FLAG peptide after ribosome display selection against monoclonal anti-V5 peptide antibodies immobilized on beads. FI = fluorescent intensity, BS = before selection, 1R = the first round of selection, 2R = the second round of selection, and 3R = the third round of selection. In the initial mRNA pool, the molar amount of mRNA of the V5 peptide of interest was $1/10^5$ times that of the FLAG peptide as its counterpart. (C) Augmentation of V5 peptide and reduction of FLAG peptide after the third round of selection. The RT-PCR products were quantified on the basis of the fluorescent band intensities in the electrophoretic gel image.

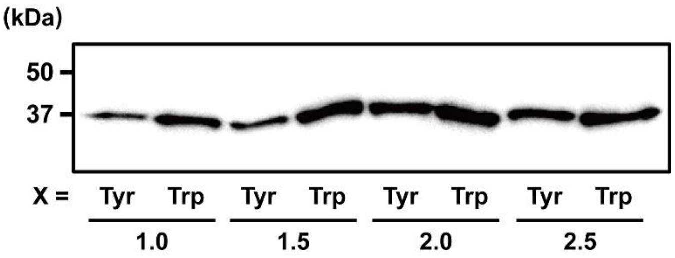

Figure S3. Western blot analysis of the expression levels of X-FLAG peptides (X = Tyr and Trp) depended on the quantity and type of synthetic X-tRNA^{UAG}. To generate X-FLAG peptides fused with a protein spacer (PS) and a hexa-histidine tag (6H), Tyr-FLAG-PS-6H and Trp-FLAG-PS-6H, in vitro translations of the mRNA encoding the sequence of UAG-FLAG-PS-6H were performed in the presence of Tyr-tRNA^{UAG} and Trp-tRNA^{UAG}, respectively.

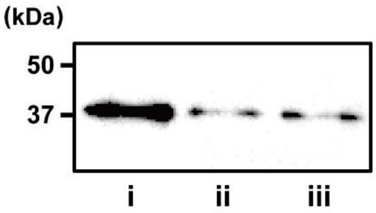

Figure S4. Western blot analysis of the expression levels of photo-cross-linkable FLAG variant peptides fused with a protein spacer (PS) and a hexa-histidine tag (6H), *p*Bzo-Phe-FLAG-PS-6H (i), *p*Bzo-Phe-FLAG(K3A)-PS-6H (ii), and *p*Bzo-Phe-FLAG(Y2A)-PS-6H (iii). To generate each peptide-fused protein, in vitro translation of the mRNA encoding the sequence of UAG-FLAG-PS-6H, UAG-FLAG(K3A)-PS-6H, or UAG-FLAG(Y2A)-PS-6H was conducted in the presence of *p*Bzo-Phe-tRNA^{UAG}.
